# Further evidence of Chelonid herpesvirus 5 (ChHV5) latency: high levels of ChHV5 DNA detected in clinically healthy marine turtles

**DOI:** 10.7717/peerj.2274

**Published:** 2016-07-27

**Authors:** Alonzo Alfaro-Núñez, Anders Miki Bojesen, Mads F. Bertelsen, Nathan Wales, George H. Balazs, M. Thomas P. Gilbert

**Affiliations:** 1 Section for Evolutionary Genomics, Center for GeoGenetics, Natural History Museum of Denmark, University of Copenhagen, Copenhagen K, Denmark; 2 Laboratorio de Biomedicina, Facultad de Ciencias de la Vida, Escuela Superior Politécnica del Litoral, Guayaquil, Ecuador; 3 Department of Veterinary Disease Biology, Veterinary Clinical Microbiology, Faculty of Health and Medical Sciences, University of Copenhagen, Frederiskberg, Copenhagen, Denmark; 4 Center for Zoo and Wild Animal Health, Copenhagen Zoo, Frederiskberg, Copenhagen, Denmark; 5 Pacific Islands Fisheries Science Center, National Marine Fisheries Service, Honolulu, HI, United States of America; 6 Trace and Environmental DNA Laboratory, School of Environment and Agriculture, Curtin University of Technology, Perth, Perth, Australia

**Keywords:** Chelonid herpesvirus 5 (ChHV5), Fibropapillomatosis (FP), Glycoprotein B, Clinically healthy, Quantitative PCR, Viral loads, Ubiquitous, Asymptomatic

## Abstract

The Chelonid herpesvirus 5 (ChHV5) has been consistently associated with fibropapillomatosis (FP), a transmissible neoplastic disease of marine turtles. Whether ChHV5 plays a causal role remains debated, partly because while FP tumours have been clearly documented to contain high concentrations of ChHV5 DNA, recent PCR-based studies have demonstrated that large proportions of asymptomatic marine turtles are also carriers of ChHV5. We used a real-time PCR assay to quantify the levels of ChHV5 Glycoprotein *B* (g*B*) DNA in both tumour and non-tumour skin tissues, from clinically affected and healthy turtles drawn from distant ocean basins across four species. In agreement with previous studies, higher ratios of viral to host DNA were consistently observed in tumour versus non-tumour tissues in turtles with FP. Unexpectedly however, the levels of ChHV5 g*B* DNA in clinically healthy turtles were significantly higher than in non-tumour tissues from FP positive turtles. Thus, a large proportion of clinically healthy sea turtle populations worldwide across species carry ChHV5 g*B* DNA presumably through persistent latent infections. ChHV5 appears to be ubiquitous regardless of the animals’ clinical conditions. Hence, these results support the theory that ChHV5 is a near ubiquitous virus with latency characteristics requiring co-factors, possibly environmental or immune related, to induce FP.

## Introduction

Historically, viral infections associated with abnormal cell growth have been reported and studied directly from tumours (Work et al., 2001). Among others, several herpesviruses can lead to tumour formation in humans (Hausen zur, 1991) and other vertebrates such as reptiles (Ariel, 2011). The marine turtle disease fibropapillomatosis (FP), has been linked to infection by the Chelonid herpesvirus 5 (ChHV5) also commonly known as the Chelonid fibropapilloma-associated herpesvirus (CFPHV) (Herbst, 1994). Despite, all attempts at culturing ChHV5 *in vitro* have been unsuccessful (Lu et al., 1999; Work et al., 2009). Thus, confirmation of ChHV5 as the primary causal agent of FP tumours remains inconclusive as defined by the four Koch’s postulates that establish relationships to identify the causative agent of a disease.

Several studies have used quantitative real time PCR (*q*PCR) methods to assay the levels of ChHV5 DNA in marine turtle tissues, in an attempt to shed further light into its potential relevance for FP. While most of such *q*PCR assays have exclusively targeted the skin tumours (Work et al., 2009), conjunctival growths, and/or tumours from visceral organs (Curry et al., 2000) sampled from FP affected turtles, non-tumour skin tissue samples of infected animals have been used to demonstrate that ChHV5 is always found at higher level in FP tumours than in healthy tissue samples from affected animals (Quackenbush et al., 2001). Although this has been interpreted as further evidence for ChHV5s role in FP, little attention has been given to the levels of ChHV5 in clinically healthy turtles, whether from feeding grounds shared with FP affected individuals or from species’ populations in which FP has never been observed. Only a handful of research studies, exclusively focused on populations historically documented with individuals infected by the FP disease, have described the detection of herpes viral DNA from clinically healthy marine turtles (Herbst et al., 2008).

In a recent series of studies we documented the presence of ChHV5 DNA sequences in a global sample set of five species of clinically healthy marine turtles, using sequenced PCR amplicons (Alfaro-Núñez & Gilbert, 2014; Alfaro-Núñez et al., 2014). In the same studies, we also investigated the possibility that these viral amplicons have been integrated in the turtle genome (Wang et al., 2013), and finding no evidence for this hypothesis, we confirmed the presence of ChHV5 DNA in healthy individuals. We did emphasise the presence of Glycoprotein *B* (g*B*) amplicons, a well-characterized protein involved in the viral cell entry, in clinically healthy turtles. This immediately begs questions as: (i) if ChHV5 is the etiological agent for FP, how can it be found in asymptomatic animals; and (ii) how do levels of ChHV5 in healthy animals compare to those originating from FP samples? To investigate this, we aimed to determine the relative concentration of ChHV5 Glycoprotein *B* (g*B*) to host nuclear DNA ratio in a sub-sample set from a recently published work (Alfaro-Núñez & Gilbert, 2014) including FP positive and negative sea turtle tissue samples using a real-time polymerase chain reaction (*q*PCR) assay in order to provide further evidence of the ChHV5 latency distinctive feature.

## Materials and Methods

### Data collection

Our dataset consisted of: ten paired tumour and non-tumour tissue samples from cases of FP in green turtles (*Chelonia mydas*) obtained from warmer climates in three geographically distant populations; Hawaii in the Pacific, Turks & Caicos in the Caribbean, and Príncipe Island off the Western coast of Africa. Five additional FP tumour and one non-tumour samples from the same populations were also included. Moreover, 16 DNA samples from four clinically healthy turtle species from other oceanic areas and several distant turtle populations were analysed: six hawksbills (*Eretmochelys imbricata*) from Principe Island and Qaru Island Kuwait; three loggerheads (*Caretta caretta*), two from Cyprus and one rehabilitated in a Danish aquarium; two leatherbacks (*Dermochelys coriacea*) from Ghana; and five DNA extracts from two green turtle individuals, one from Tortuguero (Costa Rica) and one from Denmark (four DNA extracts). This last green turtle that originated from the same previously mentioned Danish aquarium, was the only turtle that was sampled post mortem, and as such multiple skin and internal organs samples were available for analysis. In this study, a clinically healthy turtle is defined as an animal that exhibited no visual FP tumours or any other clinical signs of illness.

Combined dermis and epidermis cross sections of the surface tissue samples (5–10 mm depth) were taken from the trailing edge of the front flippers and neck of the turtles using a scalpel. A standard protocol for tissue collection was generally followed in this study (Dutton, 1996).

DNA was extracted from approximately 25 mg tissue samples using the DNeasy Tissue Kit (Qiagen, Valencia, CA, USA) following the manufacturer’s instructions. Samples were incubated with agitation for 24 h at 56 °C. The samples were then centrifuged for 5 min at 10,000 g to pellet any remaining cellular material and the supernatant was transferred into a 2 ml DNeasy Mini spin column placed in a 2 ml collection tube and centrifuged for 1 min at 8,000 g. Finally purified genomic DNA was eluted in 50 µl of Qiagen EB buffer. Extraction blanks were included to monitor for contamination. All DNA extracts and blanks were assessed and tested by singleplex PCR (Alfaro-Núñez & Gilbert, 2014) in order to check for amplification of the correct target size, and thus to ensure presence of ChHV5 and turtle DNA for quality control purposes.

All tissue skin samples were collected in strict accordance with the recommendations in the Guide for the Care and Use of Laboratory Animals of the National Institutes of Health (Eighth Edition, 2011); and exported under relevant CITES permits (host institute permit DK03). Collection of samples from Hawaii was approved by permit No: SWP I2010-03 issued by the IACUC at National Oceanic and Atmospheric Administration (NOAA). All FP tumour samples were originated from turtles that stranded with terminal FP and tissues were collected during post-mortem examination.

Approvals for the work in Turks and Caicos Islands were obtained through the Department of Environment and Maritime Affairs (DEMA). Nesting beaches (some within protected areas) were accessed with permission from landowners and DEMA. All harvested turtles were sampled as part of the legal turtle fishery with permission from fishermen, processing plants and DEMA. Samples were exported using multiple CITES permits and UK Animal Health and Veterinary Laboratories Agency permits. Permits and sample collection from Principe Island were carried out in cooperation between Zoomarine and the Faculty of Science and Technology at University of Algarve, Portugal.

Most healthy tissue samples were collected from necrotic stranded turtles. Samples from Kuwait and Costa Rica were originated from live animals following protocols approved by Kuwaiti Coast Guard and Universidad de Costa Rica (UCR) in cooperation with the Costa Rican Ministry of Environment and Energy (MINAE), respectively. Tissue sample collection followed different protocols from our various collaborators, however the majority were obtained from either skin of front flipper or neck of the turtles following the standard procedure for collection of turtle tissue samples (Dutton, 1996).

### Real-time *q*PCR assay

The DNA quantification assay was developed and tested on a Roche LightCycler 480 Real-time PCR System using SYBR Green chemistry. Although SYBR Green chemistry is less target specific than motif-specific probes such as TaqMan, we selected the former due to its greater flexibility (Wales et al., 2012), thus increasing our chances of amplifying DNA sequences of targets that could contain unknown variation (which given the paucity of comparative ChHV5 sequence available, is a distinct possibility).

Two sets of oligonucleotide primers designed to target highly conserved markers in ChHV5 Glycoprotein *B* (g*B*) gene UL27 and endogenous turtle (*nu*DNA) nuclear clone CM12 DNA were used for the *q*PCR assay (see Table 1).

**Table 1:** List of primers used in this study. List of primer sets designed and tested for ChHV5 and endogenous turtle (*nu*DNA) DNA.

Primer set	Targeted gene	Primer sequence (5′to ’3)	Length (bases)
**UL27**^a^	**Viral ChHV5 (Glycoprotein B gene UL27)**	F: CTCCGGATGGTCGCTGGC	143
		R: CTAGATACATACTGGCCRTGCTCGTC	
**nuDNA**^a^	**Turtle genomic (nuclear DNA clone CM12)**	F: ACATTGTGCTAAAAAGCAATTGTGCCT	127
		R: ACACCAGTCATGTGGAGTGGCA	

**Notes.**

a Final primer sets used in the *q*PCR assay performed accordingly to the highest quality control checks and provided the most consistent standards curves.

These two set of primers provided the most consistent standards curves (empirically 3.32 cycles in a perfectly efficient reaction by checking if each 10% dilution crosses the fluorescence threshold after the higher concentration (Wales et al., 2012). We used g*B* primer set as it has been proven more sensitive than other oligonucleotide primers available (Alfaro-Núñez & Gilbert, 2014). Each 25 µL *qPCR* reaction contained 1 U AmpliTaq Gold polymerase (Applied Biosystems, Foster City, CA, USA), 1X AmpliTaq Gold buffer, 2.5 mM MgCl_2_, 0.2 mM dNTPs, 0.4 mM primers, 1 µL 1X SYBR Green/ROX mix (Invitrogen, Carlsbad, CA, USA), and 1 µL of template DNA. Cycling conditions for the *q*PCR assay were as follows: 95.0 °C for 10 min enzyme activation, 50 cycles of 95.0 °C for 30 s, 60.0 °C for 1 min, and 72.0 °C for 1 min, followed by a melting (disassociation) curve. In order to monitor amplification dynamics and identify amplification inhibition and other experimental errors that may not be observed when only testing undiluted samples, each sample was tested in two dilution series, with a template DNA at concentrations of: 100%, 10%, and 1%; and 100%, 50% and 25%, respectively. A negative blank control was also included for all *q*PCR runs. Moreover, a series of dilutions of a standard tumour sample, obtained from our collaborators in Hawaii (Work et al., 2009) was included for every *q*PCR run, which served as positive quality control and optimized the obtained cycle threshold (C_*t*_) values (Wales et al., 2012).

In order to estimate the absolute concentration of viral g*B* and endogenous turtle nuDNA markers per µL of DNA extract in each individual specimen, a comparison was made against a well-characterized sample, *Cm-TCFP-14* (standard sample). PCR amplicons of viral g*B* and nuDNA markers were measured on a Qubit 2.0 fluorometer (Life Technologies, Carlsbad, CA) and after accounting for the specific mass of each amplicon, diluted to make standard series from 10^8^ to 10^1^ markers per µL. These standards were then used in the *q*PCR analyses as calibration for the viral g*B* and nuDNA concentrations in the different extracts. In order to assess nucleic acids loads relationship between viral g*B* and turtle nuclear DNA, the ratio of viral g*B* to turtle nuclear DNA was calculated. To test for significant differences in media levels of DNA concentrations, we used the non-parametric method Mann–Whitney–Wilcoxon test (W-t), and for the means, parametric method *t*-test (t-t) were applied to evaluate ratios dataset using r v 3.1.0 (Team, 2008). Although the starting tissue mass was only estimated to be similar between all samples, the estimated nuDNA levels were overall consistent (Fig. 2A), with the exception of exceedingly high values of clinically healthy tissue from one of the green turtles taken post mortem from an individual held in a Danish aquarium. The tissue samples were directly frozen in the absence of preservative (−18 °C) and DNA was extracted only 12 h later, which may explain the particularly high quality. However, no significant difference was seen in the nuDNA concentration between the three groups (W-t, *p* = 0.53; and t-t, *p* = 0.69). When adjusting for estimated original tissue mass and DNA extraction elute volume (50 µL DNA extract to 25 µL PCR reaction product), these figures represent 9.9^*E*+04^ (FP tumour), 3.7^*E*+01^ (FP non-tumour) and 1.0^*E*+04^ (clinically healthy animal tissue) copies per mg tissue analysed. Detailed list of samples analysed in this *q*PCR assay listed by species and population; and value results obtained are listed in Table 2 and Data S1 —Copy number loads of g*B* and nuDNA per turtle sample grouped by health status tissue.

**Table 2:** Detailed list of samples analysed in the *q*PCR assay listed by type of tissue, and providing C_*t*_ value results from the DNA quantification. List of DNA extract samples analysed for quantification of Glycoprotein *B* (g*B*) to endogenous nuclear host (nuDNA), including; sample ID, species, type of tissue, population origin, cycle threshold values per marker, estimated copy number and calculated ration g*B*/nuDNA.

Health status	Sample ID	Species	Population (sample origin)	C_*t*_ turtle nuDNA	C_*t*_ ChHV5 g*B*	nuDNA copy number	ChHV5 g*B* copy number	Ratio copy number g*B*/nuDNA
FP tumour	Cm-HaFP-11neck	*Chelonia mydas* (green)	Hawaii, USA, Northern Pacific	23.06	22.70	2,630	414	**0.16**
	Cm-HaFP-14RHF	*Chelonia mydas* (green)	Hawaii, USA, Northern Pacific	20.93	18.25	23,400	155,000	**6.62**
	Cm-HaFP-2LHF	*Chelonia mydas* (green)	Hawaii, USA, Northern Pacific	24.12	17.14	2,130	118,778	**55.76**
	Cm-HaFP-3LHF	*Chelonia mydas* (green)	Hawaii, USA, Northern Pacific	24.12	20.93	925	10,450	**11.29**
	Cm-HaFP-4neck	*Chelonia mydas* (green)	Hawaii, USA, Northern Pacific	23.89	20.98	1,073	10,120	**9.44**
	Cm-HaFP-8maxilla	*Chelonia mydas* (green)	Hawaii, USA, Northern Pacific	20.45	16.81	22,420	214,279	**9.56**
	Cm-PiFP-04	*Chelonia mydas* (green)	Principe Island, Western Africa	16.90	18.51	94,911	49,335	**0.52**
	Cm-PiFP-20	*Chelonia mydas* (green)	Principe Island, Western Africa	25.95	20.71	659	17,568	**26.67**
	Cm-PiFP-80	*Chelonia mydas* (green)	Principe Island, Western Africa	20.01	16.79	12,915	148,669	**11.51**
	Cm-PiFP-82	*Chelonia mydas* (green)	Principe Island, Western Africa	24.10	24.96	937	5,398	**5.76**
	Cm-TCFP-14	*Chelonia mydas* (green)	Turks & Caicos Islands, Caribbean Sea	20.80	18.88	8,244	56,811	**6.89**
	Cm-TCFP-2	*Chelonia mydas* (green)	Turks & Caicos Islands, Caribbean Sea	22.63	20.62	2,753	87,306	**31.71**
	Cm-TCFP-3	*Chelonia mydas* (green)	Turks & Caicos Islands, Caribbean Sea	22.57	21.80	1,144	40,962	**35.82**
	Cm-TCFP-5	*Chelonia mydas* (green)	Turks & Caicos Islands, Caribbean Sea	21.18	21.15	2,789	62,148	**22.28**
	Cm-TCFP-8	*Chelonia mydas* (green)	Turks & Caicos Islands, Caribbean Sea	27.75	26.14	21	4	**0.19**
FP non-tumoured	Cm-HaT-8	*Chelonia mydas* (green)	Hawaii, USA, Northern Pacific	21.73	24.01	10,300	72	**0.01**
	Cm-HaT-11	*Chelonia mydas* (green)	Hawaii, USA, Northern Pacific	20.04	26.80	3,900	416	**0.11**
	Cm-HaT-14	*Chelonia mydas* (green)	Hawaii, USA, Northern Pacific	20.36	28.96	10,319	61	**0.01**
	Cm-HaT-2	*Chelonia mydas* (green)	Hawaii, USA, Northern Pacific	25.16	31.39	1,093	0	**0.00**
	Cm-HaT-3	*Chelonia mydas* (green)	Hawaii, USA, Northern Pacific	23.83	30.82	1,115	18	**0.02**
	Cm-HaT-4	*Chelonia mydas* (green)	Hawaii, USA, Northern Pacific	26.54	31.13	196	15	**0.08**
	Cm-PiT-04	*Chelonia mydas* (green)	Principe Island, Western Africa	21.71	27.63	4,341	142	**0.03**
	Cm-PiT-20	*Chelonia mydas* (green)	Principe Island, Western Africa	21.04	27.26	15,357	0	**0.00**
	Cm-PiT-51	*Chelonia mydas* (green)	Principe Island, Western Africa	21.53	29.20	11,216	0	**0.00**
	Cm-PiT-80	*Chelonia mydas* (green)	Principe Island, Western Africa	20.64	26.75	7,910	0	**0.00**
	Cm-PiT-82	*Chelonia mydas* (green)	Principe Island, Western Africa	20.03	29.54	12,751	42	**0.00**
Clinically healthy	Cc-CyT-275	*Caretta caretta* (loggerhead)	Northen Cyprus, Mediterranean	23.51	23.18	1,566	16,905	**10.80**
	Cc-CyT-346	*Caretta caretta* (loggerhead)	Northen Cyprus, Mediterranean	23.06	26.84	2,090	116	**0.06**
	Cc-DkT-01	*Caretta caretta* (loggerhead)	Denmark, Danmarks Aquarium (not naturally)	23.32	23.43	1,769	14,401	**8.14**
	Cm-DkT-01liver	*Chelonia mydas* (green)	Denmark, Danmarks Aquarium (not naturally)	17.18	29.83	182,563	0	**0.00**
	Cm-DkT-01mouth	*Chelonia mydas* (green)	Denmark, Danmarks Aquarium (not naturally)	18.53	29.41	33,367	45	**0.00**
	Cm-DkT-01neck	*Chelonia mydas* (green)	Denmark, Danmarks Aquarium (not naturally)	20.34	26.93	11,959	66	**0.01**
	Cm-DkT-01rearLF	*Chelonia mydas* (green)	Denmark, Danmarks Aquarium (not naturally)	22.48	26.25	3,031	379	**0.13**
	Cm-ToT-37	*Chelonia mydas* (green)	Costa Rica, Tortuguero, Caribbean coast	24.85	25.08	663	4,998	**7.54**
	Dc-GhT-22	*Dermochelys coriacea* (leatherback)	Ghana, Western Africa	21.89	28.02	4,426	95	**0.02**
	Dc-GhT-23	*Dermochelys coriacea* (leatherback)	Ghana, Western Africa	22.63	21.95	2,753	37,205	**13.51**
	Ei-KuT-kw5	*Eretmochelys imbricata* (hawksbill)	Qaru Island, Kuwait, Persian Gulf	20.10	27.12	13,949	192	**0.01**
	Ei-KuT-kw7	*Eretmochelys imbricata* (hawksbill)	Qaru Island, Kuwait, Persian Gulf	21.29	26.30	6,503	230	**0.04**
	Ei-PiT-25	*Eretmochelys imbricata* (hawksbill)	Principe Island, Western Africa	23.84	23.02	1,267	18,732	**14.78**
	Ei-PiT-46	*Eretmochelys imbricata* (hawksbill)	Principe Island, Western Africa	23.68	22.89	1,404	20,361	**14.50**
	Ei-PiT-71	*Eretmochelys imbricata* (hawksbill)	Principe Island, Western Africa	22.49	22.11	3,012	33,577	**11.15**
	Ei-PiT-85	*Eretmochelys imbricata* (hawksbill)	Principe Island, Western Africa	24.21	23.29	1,000	15,754	**15.76**

## Results

**Figure 1:**
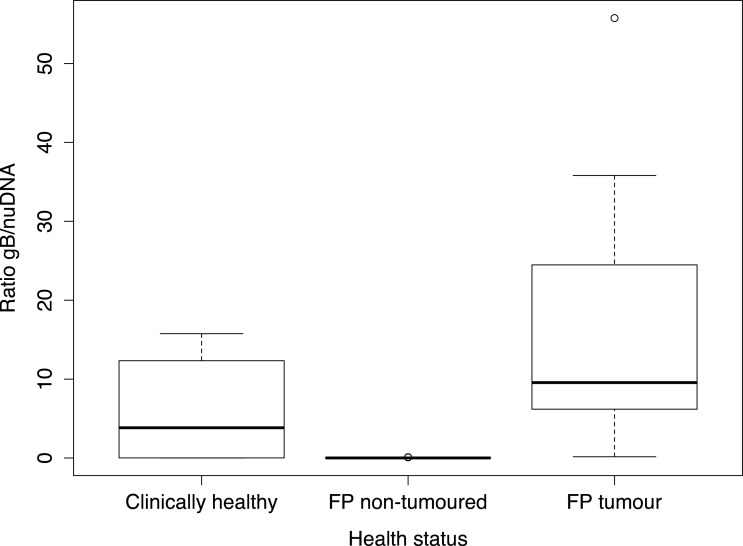
Boxplot showing viral *g* B[i] load ratios by main three different health status categories. Proportional ratio between Glycoprotein *B* (*gB*) to endogenous nuclear (nuDNA) for the main three different health status categories of sea turtle tissue samples analysed. This ratio standardises values into a range from ≤1 indicating higher nuDNA than viral *gB*; to any value ≥1 which indicates higher viral *gB* loads than endogenous nuDNA.

Consistent with previous observations (Quackenbush et al., 2001; Work et al., 2009), we observed that the highest viral loads (average ratio of 15.61, *s* ± 12.5) were in FP tumour samples, while the ratio of viral to nuclear DNA in FP non-tumour samples from FP infected animals were low to undetectable (average ratio of 0.02, *s* ± 0.00). However, tissue samples from clinically healthy animals were found to contain significantly higher viral loads of g*B* (average ratio of 6.03, W-t, *p* = 0.008, t-t, *p* = 0.002) than non-tumour samples from FP exhibiting turtles (Fig. 1).

**Figure 2:**
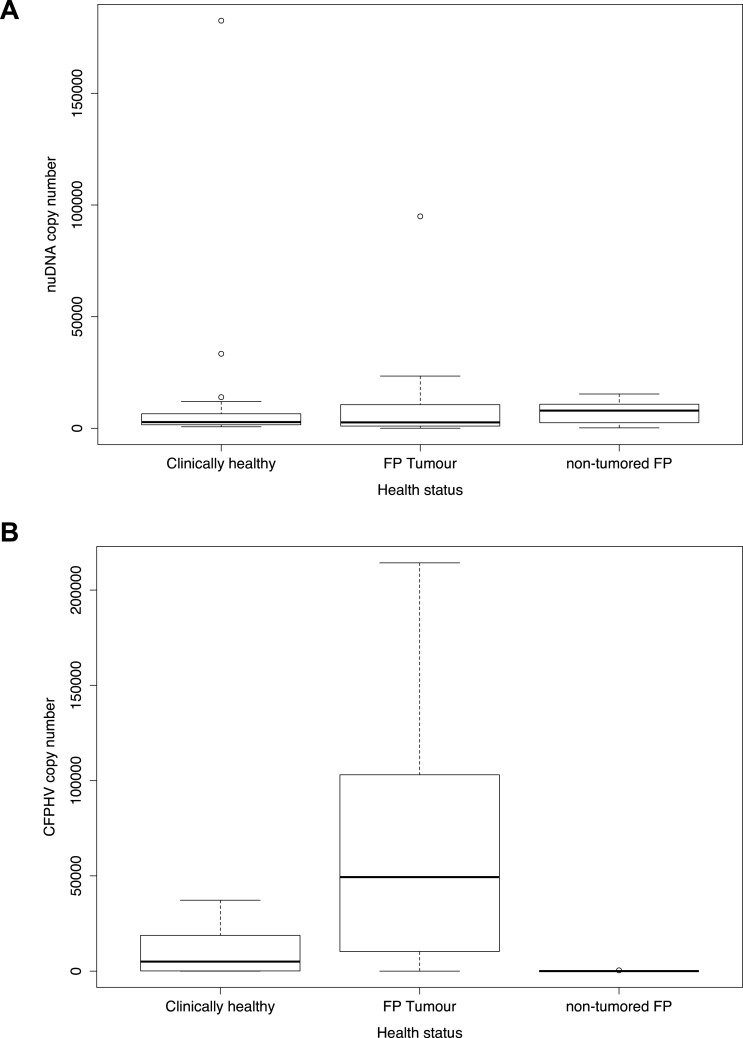
Boxplot showing g B[i] and nuDNA copy numbers by main three different health status categories. (A) Estimated copy number of endogenous nuclear DNA for the main three different health status categories of sea turtle tissue samples analysed; (B) Estimated copy number of viral Glycoprotein B[i] according to the main three different health status categories of sea turtle tissue samples analysed.

Although the starting tissue mass was only estimated to be similar between all samples, the estimated nuDNA levels were overall consistent (Fig. 2A). Based on standard curve, we estimate that the median of g*B* is approximately 4.9^*E*+04^ copies/µL DNA extract in FP tumours, 1.8^*E*+01^ copies/µL in non-tumour tissue from FP animals and 5.8^*E*+04^ copies/µL in clinically healthy turtles (Fig. 2B).

## Discussion

ChHV5 has been associated as primary etiological agent of FP by three lines of evidence. First, every tumour analysed by polymerase chain reaction (PCR) has yielded ChHV5 sequences (Herbst et al., 2004; Alfaro-Núñez & Gilbert, 2014), second, tumours occasionally show epidermal viral inclusions with ultrastructural similar to herpesvirus-like particles (Jacobson et al., 1991); and third, experimental transmission of the disease using cell-free tumour extracts inoculated into healthy turtles resulted in the development of FP disease (Herbst et al., 1995). Nevertheless, the role of ChHV5 as causal agent for FP remains inconclusive as; (i) *in vitro* culturing has been repetitively unsuccessful and (ii) ChHV5 DNA can be also found in asymptomatic turtles (Alfaro-Núñez & Gilbert, 2014).

The observation of higher levels of ChHV5 DNA in tumour versus non-tumour samples from FP exhibiting turtles was expected, as ChHV5 DNA burdens are increased within tumour tissues (see Table 2 and Data S1 —copy number loads of g*B* and nuDNA per turtle sample grouped by health status tissue). What is remarkable though, is that on average, clinically healthy turtle samples exhibited viral to host DNA ratios that were significantly higher than those within non-tumour affected tissues of FP exhibiting turtles (Fig. 2B). A key fact in this regard is that no visually observable FPs were reported for any of the ‘healthy’ turtles sampled during the tissue collection. Although ChHV5 DNA has previously been detected in skin samples from clinically healthy green, loggerhead and olive ridley turtles (Herbst et al., 2008), these animals were from populations historically well documented containing FP infected turtles.

We reiterate that the small number of samples analysed in this study represent a sub-sample set from a larger number of turtle populations recently published work (Alfaro-Núñez & Gilbert, 2014). Thus, all samples analysed here have been already detected viral DNA positive by PCR providing further evidence of actual presence of the herpesvirus. Our results however provide first evidence of viral loads in multiple sub-samples of clinically affected and healthy turtles from distant ocean basins across four species. In fact, the ratios of g*B* to turtle nuDNA were actually very high (>10) in six of the clinically healthy turtles (Fig. 1). These samples correspond to four hawksbill turtles from Principe Island, Western Africa, which are known to share feeding grounds with green turtle FP exhibiting populations (Duarte et al., 2012); one leatherback from Ghana and one loggerhead in the Mediterranean (Table 2). FP has rarely been documented in hawksbill (Herbst, 1994; D’Amato & Moraes-Neto, 2000) and leatherback (Huerta et al., 2000) species, so the detection of ChHV5 g*B* DNA in these two species represent a quandary. Nuclear DNA levels were consistent among samples and routine use of negative and positive controls performed as expected, therefore, it is not suspected that these results are artefact of procedure. Furthermore, as with most alphaherpesviruses ChHV5 primarily appears to target skin tissue (Kang et al., 2008). Thus, the presence of ChHV5 g*B* DNA in skin from clinically healthy animals may represent an early stage of infection which remains in latency, and detection of viral concentrations in tumour-free animals may be a means of identifying animals that will eventually develop FP tumours (Quackenbush et al., 2001) subjected to the appropriate co-factors triggering the infection. The mechanism underlying this phenomenon remains unclear and was not addressed in this study. There is no evidence that immunosuppression is needed for FP development, but there is evidence that FP does lead to immunosuppression in Hawaiian green turtles (Work et al., 2001).

The g*B* gene selected for this study is an envelope protein that is only found in large amounts in intact virions undergoing lytic replication (Zhu et al., 2004). As in FP in turtles, skin tumours are the only tissues where lytic replication with intact virions has been consistently documented (Work et al., 2009), one would expect g*B* levels to be relatively high in these tissues. Thus, we were anticipating and expecting high levels of ChHV5 g*B* DNA in FP tumours, followed by relatively low in FP non-tumours and extremely low to absent in healthy tissue samples. However, high levels of herpesviral DNA in skin from clinically healthy animals suggest another not described process in regards to development of ChHV5 latency.

Many herpesviruses are maintained in sensory neurons in a latent state in which no clinical disease is produced and characterised by the repression of most viral lytic cycle genes and the abundant expression of certain types of viral RNA (Kramer et al., 1998). Such repression of viral protein synthesis during latency has led to the general view of latency as being a quiescent and antigenically silent infection that is ignored by host immunity (Ramachandran et al., 2010). However, very low levels of gene transcripts and proteins from all kinetic classes of herpes simplex virus have been detected in latently laboratory infected rodents (Feldman et al., 2002). Previous studies have detected nucleosomes on the herpes simplex virus (HSV) genome during a lytic infection (Deshmane & Fraser, 1989) that are not arranged in an equally spaced array like in cellular DNA. However, as in cellular DNA, the promoter regions of several viral genes have been shown to be associated with nucleosomes containing modified histone proteins that are generally found associated with actively transcribed genes (Oh & Fraser, 2008). Because no histones were detected inside HSV type 1 capsids, the viral genome probably starts to associate with histones after being transported from infecting virions into the host nucleus. This represents evidence that a herpesvirus genome can remain intact in the host nucleus disassociated from the viral envelope protection. Thus, we propose that a similar situation exists in turtles latently infected with ChHV5, where our data provides evidence of g*B* DNA being consistently present in clinically healthy marine turtles across four different species. We caution here that our methods detect only a portion of the g*B* DNA and does not imply production of lytic virus in normal skin from FP free animals.

While Koch’s postulates are still regarded as the most compelling proof of causation, there are documented instances where disease agents cannot be cultured, yet are now known to be causative (Lipkin, 2009), such as the herpes simplex and polio viruses. Although a 21st century adaptation of Koch’s postulates (Fredericks & Relman, 1995) have been proposed to reflect the introduction of culture-independent molecular methods, these updated criteria are also not yet fulfilled by ChHV5. Thus, ChHV5’s association with FP remains tantalising, yet unsolved.

In summary, our findings offer evidence that clinically healthy marine turtles from distant sites worldwide where FP has not been reported yet across species carry ChHV5 DNA through persistent latent infections, often at very high levels. This in turn provides additional evidence for hypotheses (Herbst & Klein, 1995; Van Houtan, Hargrove & Balazs, 2010) that ChHV5 is a near ubiquitous virus and alone does not cause infection, but requires one or more possibly environmental or immune related co-factors or triggers to induce FP.

##  Supplemental Information

10.7717/peerj.2274/supp-1 Data S1 Copy number loads of g B[i] and nuDNA per turtle sample grouped by health status tissue Copy number loads of viral Glycoprotein B *(g B*) and endogenous turtle-host nuclear (nuDNA) per each individual sample grouped by health status type of tissue-values presented in log_10_ scale.

10.7717/peerj.2274/supp-2 Table S1

## Additional Information and Declarations

### Data Availability

The following information was supplied regarding data availability: The raw data has been supplied as Data S1.

## References

[ref-1] Alfaro-Núñez A, Frost Bertelsen M, Bojesen A, Rasmussen I, Zepeda-Mendoza L, Tange Olsen M, Gilbert M (2014). Global distribution of Chelonid fibropapilloma-associated herpesvirus among clinically healthy sea turtles. BMC Evolutionary Biology.

[ref-2] Alfaro-Núñez A, Gilbert MTP (2014). Validation of a sensitive PCR assay for the detection of Chelonid fibropapilloma-associated herpesvirus in latent turtle infections. Journal of Virological Methods.

[ref-3] Ariel E (2011). Viruses in reptiles. Veterinary Research.

[ref-4] Curry SS, Brown DR, Gaskin JM, Jacobson ER, Ehrhart LM, Blahak S, Herbst LH, Klein PA (2000). Persistent infectivity of a disease-associated herpesvirus in green turtles after exposure to seawater. Journal of Wildlife Diseases.

[ref-5] D’Amato AF, Moraes-Neto M (2000). First documentation of fibropapillomas verified by histopathology in *Eretmochelys imbricata*. Marine Turtle Newsletter.

[ref-6] Deshmane SL, Fraser NW (1989). During latency, herpes simplex virus type 1 DNA is associated with nucleosomes in a chromatin structure. Journal of Virology.

[ref-7] Duarte A, Faısca P, Loureiro NS, Rosado R, Gil S, Pereira N, Tavares L (2012). First histological and virological report of fibropapilloma associated with herpesvirus in *Chelonia mydas* at Prıncipe Island, West Africa. Archives of Virology.

[ref-8] Dutton PH, Bowen BW, Witzell WN (1996). Methods for collection and preservation of samples for sea turtle genetic studies. Proceedings of the international symposium on sea turtle conservation genetics.

[ref-9] Feldman LT, Ellison AR, Voytek CC, Yang L, Krause P, Margolis TP (2002). Spontaneous molecular reactivation of herpes simplex virus type 1 latency in mice. Proceedings of the National Academy of Sciences of the United States of America.

[ref-10] Fredericks DN, Relman DA (1995). Sequence-based identification of microbial pathogens: a reconsideration of Koch’s postulates. Clinical Microbiology Reviews.

[ref-11] Hausen zur H (1991). Viruses in human cancers. Science.

[ref-12] Herbst LH (1994). Fibropapillomatosis of marine turtles. Annual Review of fish Diseases.

[ref-13] Herbst LH, Ene A, Su M, DeSalle R, Lenz J (2004). Tumor outbreaks in marine turtles are not due to recent herpesvirus mutations. Current Biology.

[ref-14] Herbst LH, Jacobson ER, Moretti RH, Brown T, Sundberg JP, Klein PA (1995). Experimental transmission of green turtle fibropapillomatosis using cell-free tumor extracts. Diseases of Aquatic Organisms.

[ref-15] Herbst LH, Klein PA (1995). Green turtle fibropapillomatosis—challenges to assessing the role of environmental cofactors. Environmental Health Perspectives.

[ref-16] Herbst LH, Lemaire S, Ene AR, Heslin DJ, Ehrhart LM, Bagley DA, Klein PA, Lenz J (2008). Use of baculovirus-expressed glycoprotein H in an enzyme-linked immunosorbent assay developed to assess exposure to chelonid fibropapillomatosis-associated herpesvirus and its relationship to the prevalence of fibropapillomatosis in sea turtles. Clinical and Vaccine Immunology.

[ref-17] Huerta P, Pineda H, Aguirre AA, Spraker TR, Barragan A (2000). First confirmed case of fibropapilloma in a leatherback turtle (*Dermochelys coriacea*). http://www.nmfs.noaa.gov/pr/pdfs/species/turtlesymposium2000.pdf.

[ref-18] Jacobson ER, Buergett C, Williams B, Harris RK (1991). Herpesvirus in cutaneous fibropapillomas of the green turtle *Chelonia mydas*. Diseases of Aquatic Organisms.

[ref-19] Kang KI, Torres-Velez FJ, Zhang J, Moore PA, Moore DP, Rivera S, Brown CC (2008). Localization of fibropapilloma-associated turtle herpesvirus in green turtles (*Chelonia mydas*) by in-situ hybridization. Journal of Comparative Pathology.

[ref-20] Kramer MF, Chen S-H, Knipe DM, Coen DM (1998). Accumulation of viral transcripts and DNA during establishment of latency by herpes simplex virus. Journal of Virology.

[ref-21] Lipkin WI (2009). Microbe hunting in the 21st century. Proceedings of the National Academy of Sciences of the United States of America.

[ref-22] Lu YN, Nerurkar VR, Aguirre AA, Work TM, Balazs GH, Yanagihara R (1999). Establishment and characterization of 13 cell lines from a green turtle (*Chelonia mydas*) with fibropapillomas. In Vitro Cellular and Developmental Biology—Animal.

[ref-23] Oh J, Fraser NW (2008). Temporal association of the herpes simplex virus genome with histone proteins during a lytic infection. Journal of Virology.

[ref-24] Quackenbush SL, Casey RN, Murcek RJ, Paul TA, Work TM, Limpus CJ, Chaves A, duToit L, Perez JV, Aguirre AA, Spraker TR, Horrocks JA, Vermeer LA, Balazs GH, Casey JW (2001). Quantitative analysis of herpesvirus sequences from normal tissue and fibropapillomas of marine turtles with real-time PCR. Virology.

[ref-25] Ramachandran S, Davoli KA, Yee MB, Hendricks RL, Kinchington PR (2010). Delaying the expression of herpes simplex virus type 1 glycoprotein *B*(*gB*) to a true late gene alters neurovirulence and inhibits the *gB*− CD8 + T-cell response in the trigeminal ganglion. Journal of Virology.

[ref-26] Team RDC (2008). Team, R Development Core (2008). R: a language and environment for statistical computing.

[ref-27] Van Houtan KS, Hargrove SK, Balazs GH (2010). Land use, macroalgae, and a tumor-forming disease in marine turtles. PLoS ONE.

[ref-28] Wales N, Alberto Romero-Navarro J, Cappellini E, Gilbert MTP (2012). Choosing the best plant for the job: a cost-effective assay to prescreen ancient plant remains destined for shotgun sequencing. PLoS ONE.

[ref-29] Wang Z, Pascual-Anaya J, Zadissa A, Li W, Niimura Y, Huang Z, Li C, White S, Xiong Z, Fang D, Wang B, Ming Y, Chen Y, Zheng Y, Kuraku S, Pignatelli M, Herrero J, Beal K, Nozawa M, Li Q, Wang J, Zhang H, Yu L, Shigenobu S, Wang J, Liu J, Flicek P, Searle S, Wang J, Kuratani S, Yin Y, Aken B, Zhang G, Irie N (2013). The draft genomes of soft-shell turtle and green sea turtle yield insights into the development and evolution of the turtle-specific body plan. Nature Genetics.

[ref-30] Work TM, Dagenais J, Balazs GH, Schumacher J, Lewis TD, Leong J-AC, Casey RN, Casey JW (2009). *In vitro* biology of fibropapilloma-associated turtle herpesvirus and host cells in Hawaiian green turtles (*Chelonia mydas*). Journal of General Virology.

[ref-31] Work TM, Rameyer RA, Balazs GH, Cray C, Chang SP (2001). Immune status of free-ranging green turtles with fibropapillomatosis from Hawaii. Journal of Wildlife Diseases.

[ref-32] Zhu FX, Chong JM, Wu L, Yuan Y (2004). Virion proteins of Kaposi’s sarcoma-associated herpesvirus. Journal of Virology.

