# Supplementary material for: Further evidence of Chelonid herpesvirus 5 (ChHV5) latency: high levels of ChHV5 DNA detected in clinically healthy marine turtles
Source: PeerJ. 2016 Jul 27;4:e2274. doi: 10.7717/peerj.2274 (PMC4974929; doi:10.7717/peerj.2274)
Supplement: Data S1 — Copy number loads of viral Glycoprotein B (g B) and endogenous turtle-host nuclear (nuDNA) per each individual sample grouped by health status type of tissue-values presented in log10 scale. [file peerj-04-2274-s001.pdf]

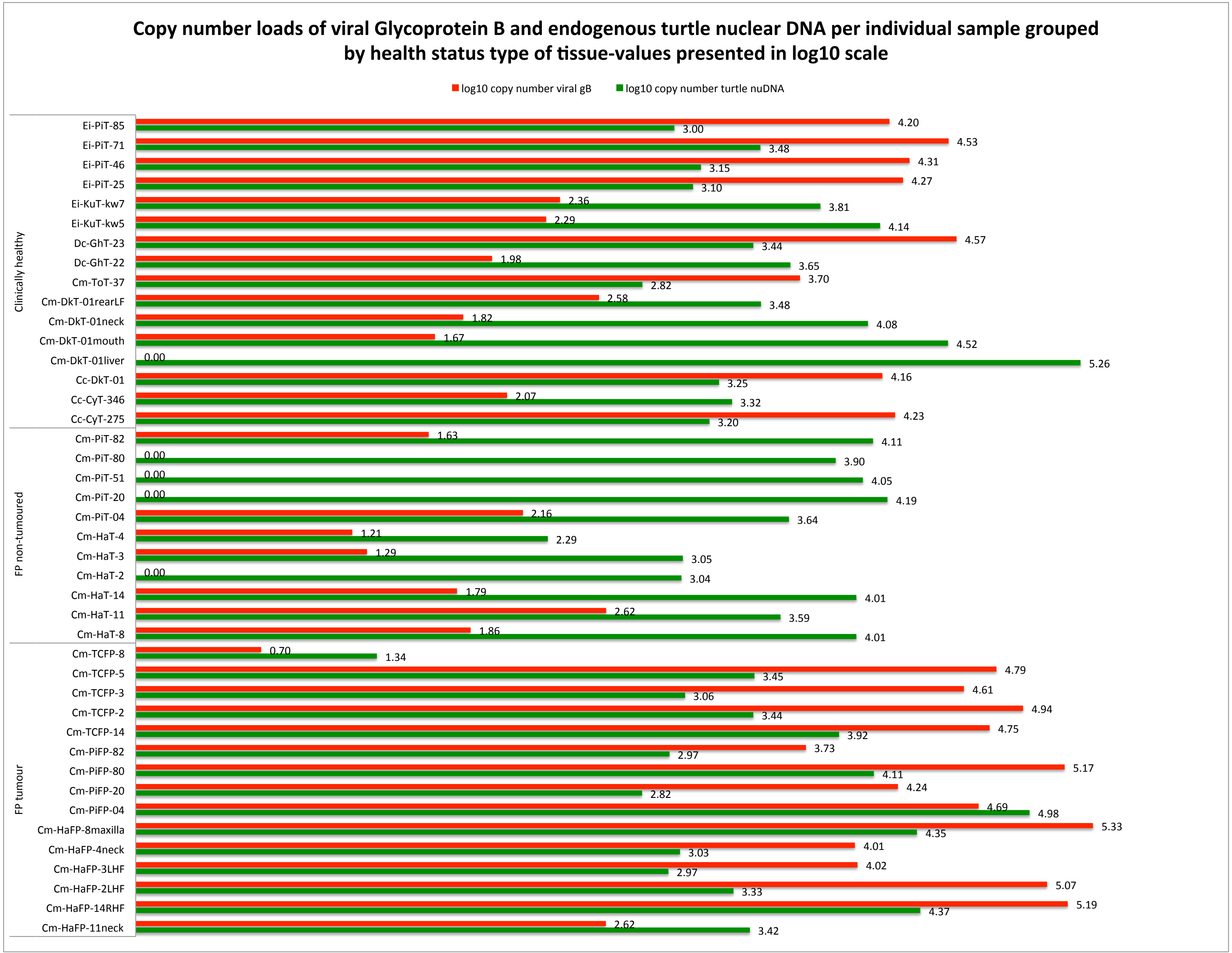
Copy number loads of viral Glycoprotein B and endogenous turtle nuclear DNA per individual sample grouped by health status type of tissue-values presented in log10 scale
log10 copy number viral gB
log10 copy number turtle nuDNA
Clinically healthy
FP non-tumoured
FP tumour
Ei-PiT-85 4.20 3.00
Ei-PiT-71 4.53 3.48
Ei-PiT-46 4.31 3.15
Ei-PiT-25 4.27 3.10
Ei-KuT-kw7 2.36 3.81
Ei-KuT-kw5 2.29 4.14
Dc-GhT-23 4.57 3.44
Dc-GhT-22 1.98 3.65
Cm-ToT-37 3.70 2.82
Cm-DkT-01rearLF 2.58 3.48
Cm-DkT-01neck 1.82 4.08
Cm-DkT-01mouth 1.67 4.52
Cm-DkT-01liver 0.00 5.26
Cc-DkT-01 4.16 3.25
Cc-CyT-346 2.07 3.32
Cc-CyT-275 4.23 3.20
Cm-PiT-82 1.63 4.11
Cm-PiT-80 0.00 3.90
Cm-PiT-51 0.00 4.05
Cm-PiT-20 0.00 4.19
Cm-PiT-04 2.16 3.64
Cm-HaT-4 1.21 2.29
Cm-HaT-3 1.29 3.05
Cm-HaT-2 0.00 3.04
Cm-HaT-14 1.79 4.01
Cm-HaT-11 2.62 3.59
Cm-HaT-8 1.86 4.01
Cm-TCFP-8 0.70 1.34
Cm-TCFP-5 4.79 3.45
Cm-TCFP-3 4.61 3.06
Cm-TCFP-2 4.94 3.44
Cm-TCFP-14 4.75 3.92
Cm-PiFP-82 3.73 2.97
Cm-PiFP-80 5.17 4.11
Cm-PiFP-20 4.24 2.82
Cm-PiFP-04 4.69 4.98
Cm-HaFP-8maxilla 5.33 4.35
Cm-HaFP-4neck 4.01 3.03
Cm-HaFP-3LHF 4.02 2.97
Cm-HaFP-2LHF 5.07 3.33
Cm-HaFP-14RHF 5.19 4.37
Cm-HaFP-11neck 2.62 3.42
