# Supplementary material for: Further evidence of Chelonid herpesvirus 5 (ChHV5) latency: high levels of ChHV5 DNA detected in clinically healthy marine turtles
Source: PeerJ. 2016 Jul 27;4:e2274. doi: 10.7717/peerj.2274 (PMC4974929; doi:10.7717/peerj.2274)
Supplement: Table S1 [file peerj-04-2274-s002.pdf]

Supplementary Table S2. List of samples analysed Fibropapillomatosis tumoured and non-tumoured green turtles (Chelonia mydas) from three confirmed infected populations. Moreover, non-tumoured samples from four more turtle species are also included for the quality-checking and analysis of qPCR Ct values

Health Status	sample ID	Species	population (sample origin)	C_t turtle nuclear DNA	C_t virus gene UL27	Delta (Δ) C_t = virus UL27 - nu DNA	nuDNA copy number	CFPHV copy number
FP tumour	Cm-HaFP-11neck	*Chelonia mydas* (green)	Hawaii, USA, Northern Pacific	23,06	22,7	-0,36	2,63E+03	4,14E+02
	Cm-HaFP-14RHF	*Chelonia mydas* (green)	Hawaii, USA, Northern Pacific	20,93	18,25	-2,68	2,34E+04	1,55E+05
	Cm-HaFP-2LHF	*Chelonia mydas* (green)	Hawaii, USA, Northern Pacific	24,12	17,14	-6,98	2,13E+03	1,19E+05
	Cm-HaFP-3LHF	*Chelonia mydas* (green)	Hawaii, USA, Northern Pacific	24,12	20,93	-3,19	9,25E+02	1,05E+04
	Cm-HaFP-4neck	*Chelonia mydas* (green)	Hawaii, USA, Northern Pacific	23,89	20,98	-2,91	1,07E+03	1,01E+04
	Cm-HaFP-8maxilla	*Chelonia mydas* (green)	Hawaii, USA, Northern Pacific	20,45	16,81	-3,64	2,24E+04	2,14E+05
	Cm-PiFP-04	*Chelonia mydas* (green)	Principe Island, Western Africa	16,9	18,51	1,61	9,49E+04	4,93E+04
	Cm-PiFP-20	*Chelonia mydas* (green)	Principe Island, Western Africa	25,95	20,71	-5,24	6,59E+02	1,76E+04
	Cm-PiFP-80	*Chelonia mydas* (green)	Principe Island, Western Africa	20,01	16,79	-3,22	1,29E+04	1,49E+05
	Cm-PiFP-82	*Chelonia mydas* (green)	Principe Island, Western Africa	24,1	24,96	0,86	9,37E+02	5,40E+03
	Cm-TCFP-14	*Chelonia mydas* (green)	Turks & Caicos Islands, Caribbean Sea	20,8	18,88	-1,92	8,24E+03	5,68E+04
	Cm-TCFP-2	*Chelonia mydas* (green)	Turks & Caicos Islands, Caribbean Sea	22,63	20,62	-2,01	2,75E+03	8,73E+04
	Cm-TCFP-3	*Chelonia mydas* (green)	Turks & Caicos Islands, Caribbean Sea	22,57	21,8	-0,77	1,14E+03	4,10E+04
	Cm-TCFP-5	*Chelonia mydas* (green)	Turks & Caicos Islands, Caribbean Sea	21,18	21,15	-0,03	2,79E+03	6,21E+04
	Cm-TCFP-8	*Chelonia mydas* (green)	Turks & Caicos Islands, Caribbean Sea	27,75	26,14	-1,61	2,10E+01	4,00E+00
non-tumoured FP	Cm-HaT-8	*Chelonia mydas* (green)	Hawaii, USA, Northern Pacific	21,73	24,01	2,28	1,03E+04	7,22E+01
	Cm-HaT-11	*Chelonia mydas* (green)	Hawaii, USA, Northern Pacific	20,04	26,8	6,76	3,90E+03	4,16E+02
	Cm-HaT-14	*Chelonia mydas* (green)	Hawaii, USA, Northern Pacific	20,36	28,96	8,6	1,03E+04	6,06E+01
	Cm-HaT-2	*Chelonia mydas* (green)	Hawaii, USA, Northern Pacific	25,16	31,39	6,23	1,09E+03	4,00E-05
	Cm-HaT-3	*Chelonia mydas* (green)	Hawaii, USA, Northern Pacific	23,83	30,82	6,99	1,11E+03	1,84E+01
	Cm-HaT-4	*Chelonia mydas* (green)	Hawaii, USA, Northern Pacific	26,54	31,13	4,59	1,96E+02	1,51E+01
	Cm-PiT-04	*Chelonia mydas* (green)	Principe Island, Western Africa	21,71	27,63	5,92	4,34E+03	1,42E+02
	Cm-PiT-20	*Chelonia mydas* (green)	Principe Island, Western Africa	21,04	27,26	6,22	1,54E+04	0,00E+00
	Cm-PiT-51	*Chelonia mydas* (green)	Principe Island, Western Africa	21,53	29,2	7,67	1,12E+04	4,18E-04
	Cm-PiT-80	*Chelonia mydas* (green)	Principe Island, Western Africa	20,64	26,75	6,11	7,91E+03	0,00E+00
	Cm-PiT-82	*Chelonia mydas* (green)	Principe Island, Western Africa	20,03	29,54	9,51	1,28E+04	4,18E+01
Clinically healthy	Cc-CyT-275	*Caretta caretta* (loggerhead)	Northen Cyprus, Mediterranean	23,51	23,18	-0,33	1,57E+03	1,69E+04
	Cc-CyT-346	*Caretta caretta* (loggerhead)	Northen Cyprus, Mediterranean	23,06	26,84	3,78	2,09E+03	1,16E+02
	Cc-DkT-01	*Caretta caretta* (loggerhead)	Denmark, Danmarks Aquarium (not naturally)	23,32	23,43	0,11	1,77E+03	1,44E+04
	Cc-DkT-02	*Caretta caretta* (loggerhead)	Denmark, Danmarks Aquarium (not naturally)	23,53	22,58	-0,95	1,77E+03	2,48E+04
	Cm-DkT-01liver	*Chelonia mydas* (green)	Denmark, Danmarks Aquarium (not naturally)	17,18	29,83	12,65	1,83E+05	2,79E-04
	Cm-DkT-01mouth	*Chelonia mydas* (green)	Denmark, Danmarks Aquarium (not naturally)	18,53	29,41	10,88	3,34E+04	4,54E+01
	Cm-DkT-01neck	*Chelonia mydas* (green)	Denmark, Danmarks Aquarium (not naturally)	20,34	26,93	6,59	1,20E+04	6,57E+01
	Cm-DkT-01rearLF	*Chelonia mydas* (green)	Denmark, Danmarks Aquarium (not naturally)	22,48	26,25	3,77	3,03E+03	3,79E+02
	Cm-ToT-37	*Chelonia mydas* (green)	Costa Rica, Tortuguero, Caribbean coast	24,85	25,08	0,23	6,63E+02	5,00E+03
	Dc-GhT-22	*Dermochelys coriacea* (leatherback)	Ghana, Western Africa	21,89	28,02	6,13	4,43E+03	9,54E+01
	Dc-GhT-23	*Dermochelys coriacea* (leatherback)	Ghana, Western Africa	22,63	21,95	-0,68	2,75E+03	3,72E+04
	Ei-KuT-kw5	*Eretmochelys imbricata* (hawksbill)	Qaru Island, Kuwait, Persian Gulf	20,1	27,12	7,02	1,39E+04	1,92E+02
	Ei-KuT-kw7	*Eretmochelys imbricata* (hawksbill)	Qaru Island, Kuwait, Persian Gulf	21,29	26,3	5,01	6,50E+03	2,30E+02
	Ei-PiT-25	*Eretmochelys imbricata* (hawksbill)	Principe Island, Western Africa	23,84	23,02	-0,82	1,27E+03	1,87E+04
	Ei-PiT-46	*Eretmochelys imbricata* (hawksbill)	Principe Island, Western Africa	23,68	22,89	-0,79	1,40E+03	2,04E+04
	Ei-PiT-71	*Eretmochelys imbricata* (hawksbill)	Principe Island, Western Africa	22,49	22,11	-0,38	3,01E+03	3,36E+04
	Ei-PiT-85	*Eretmochelys imbricata* (hawksbill)	Principe Island, Western Africa	24,21	23,29	-0,92	1,00E+03	1,58E+04
